# miR-92b-3p-TSC1 axis is critical for mTOR signaling-mediated vascular smooth muscle cell proliferation induced by hypoxia

**DOI:** 10.1038/s41418-018-0243-z

**Published:** 2018-12-05

**Authors:** Jihui Lee, Jeongyeon Heo, Hara Kang

**Affiliations:** Division of Life Sciences, College of Life Sciences and Bioengineering, Incheon National University, Incheon, 406-772 Republic of Korea

**Keywords:** RNA, Epigenetics, Cardiovascular diseases

## Abstract

Pulmonary artery smooth muscle cells (PASMCs) undergo proliferation by the mammalian target of rapamycin (mTOR) signaling pathway under hypoxia. Hypoxia induces expression of a specific set of microRNAs (miRNAs) in a variety of cell types. We integrated genomic analyses of both small non-coding RNA and coding transcripts using next-generation sequencing (NGS)-based RNA sequencing with the molecular mechanism of the mTOR signaling pathway in hypoxic PASMCs. These analyses revealed hypoxia-induced miR-92b-3p as a potent regulator of the mTOR signaling pathway. We demonstrated that miR-92b-3p directly targets the 3′-UTR of a negative regulator in the mTOR signaling pathway, TSC1. mTOR signaling and consequent cell proliferation were promoted by enforced expression of miR-92b-3p but inhibited by knocking down endogenous miR-92b-3p. Furthermore, inhibition of miR-92b-3p attenuated hypoxia-induced proliferation of vascular smooth muscle cells (VSMCs). Therefore, this study elucidates a novel role of miR-92b-3p as a hypoxamir in the regulation of the mTOR signaling pathway and the pathological VSMC proliferative response under hypoxia. These findings will help us better understand the miRNA-mediated molecular mechanism of the proliferative response of hypoxic VSMCs through the mTOR signaling pathway.

## Introduction

Hypoxia contributes to the pathogenesis of various human diseases, including cancer, stroke and pulmonary artery hypertension [1, 2]. Hypoxia stimulates abnormal proliferation and migration of vascular smooth muscle cells (VSMCs), resulting in decreased luminal diameter and obstruction of pulmonary arteries [3]. Although the molecular mechanisms involved in the proliferative and migratory responses are still not completely understood, it has been shown that chronic hypoxia-induced proliferation requires activation of the mTOR signaling pathway [4]. The potential importance of the mTOR signaling pathway for regulating proliferation and survival of VSMCs and in development of vascular remodeling in pulmonary hypertension has been suggested [5].

mTOR is a well-conserved serine/threonine kinase that plays a central role in the signaling network controlling cell proliferation, growth, survival and metabolism in response to various environmental cues [6]. mTOR belongs to the phosphoinositide 3-kinase (PI3K)-related kinase family and interacts with several proteins to form two distinct complexes named mTOR complex 1 (mTORC1) and 2 (mTORC2). mTORC1 regulates cell growth through S6K1 and 4E-BP1 in response to nutrients and growth factors. A heterodimer consisting of tuberous sclerosis 1 (TSC1) and TSC2 is a key upstream regulator of mTORC1 and functions as a GTPase-activating protein (GAP) for the Ras homolog enriched in brain (RHEB) GTPase. GTP-bound RHEB interacts with mTORC1 and activates the mTOR signaling pathway. As a RHEB GAP, TSC1/2 negatively regulates the mTOR signaling pathway.

Hypoxia regulates gene expression through a transcription factor, hypoxia-inducible factor-1 (HIF-1), which orchestrates the transcriptional regulation of a variety of genes such as vascular endothelial growth factor (VEGF) [7]. Hypoxia also regulates the expression of a specific group of miRNAs, termed hypoxamirs, through transcriptional and non-transcriptional mechanisms. The master hypoxamir, miR-210, is upregulated at the transcriptional level by HIF-1 in a variety of cell types in response to hypoxia [8]. Upregulation of miR-210 has been detected especially in cardiovascular diseases and solid tumors [9]. In addition to transcriptional regulation of miRNAs, hypoxia can regulate miRNA levels through post-translational modification of a component of the RNA-induced silencing complex, Argonaute2 (Ago2), independently of HIF-1. Hypoxia induces prolyl-hydroxylation and accumulation of Ago2, leading to increased expression levels of miRNAs such as miR-451 [10].

There is growing evidence that miRNAs play important roles in cellular responses to hypoxia and in pulmonary hypertensive vascular remodeling [11–14]. Furthermore, several miRNAs that regulate the mTOR signaling pathway have been identified in various cell types. For example, miR-96 and miR-199a-3p target mTOR in prostate cancer cells and hepatocellular carcinoma, respectively [15, 16]. miR-155 also regulates the mTOR signaling pathway by suppressing multiple targets such as RHEB and Rictor [17]. Therefore, we hypothesized that hypoxia modulates miRNA expression to activate the mTOR signaling pathway, leading to a pathological VSMC proliferative response.

Here, we present the miRNA expression profile of hypoxia-induced pulmonary artery smooth muscle cells (PASMCs) in comparison with that of normoxia cells by NGS-based small RNA sequencing. We observed unique miRNA expression signatures that could be regulators under low oxygen stress. We provide evidence for a major contribution of miR-92b-3p to the regulation of the hypoxia-mediated mTOR signaling pathway and identify TSC1 as a direct target of miR-92b-3p. Our finding of the miR-92b-3p-TSC1 axis, specifically induced by hypoxia, will help us better understand the molecular mechanism of the proliferative response of VSMCs through the mTOR signaling pathway. Furthermore, the importance of miR-92b-3p modulation to mTOR signaling-mediated cellular responses will provide invaluable insight for therapeutic approaches to abnormal VSMC proliferation, triggering vascular remodeling in hypoxia-induced pulmonary hypertension.

## Results

### Hypoxia induces mTOR signaling pathway and promotes proliferation of vascular smooth muscle cells

**Fig. 1:**
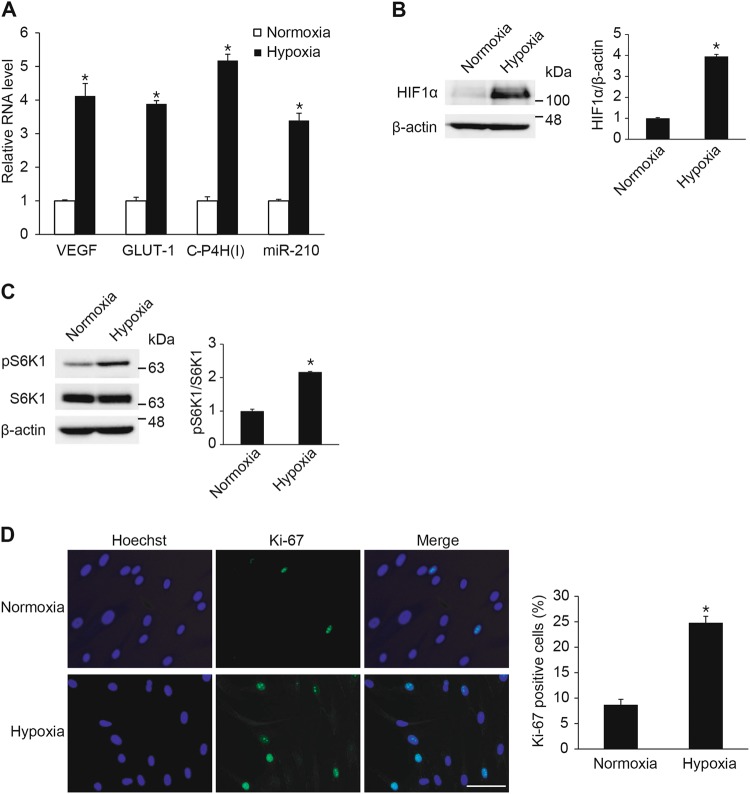
Hypoxia promotes VSMC proliferation through the mTOR signaling pathway. **a** PASMCs were exposed to normoxia or hypoxia for 24 h and subjected to qRT-PCR analysis of VEGF, GLUT-1, C-P4H(I) and miR-210. The relative levels of mRNA expression normalized to 18S rRNA were quantitated. The expression levels of miR-210 were normalized to U6 snRNA. *p < 0.05. **b** Total cell lysates from PASMCs exposed to normoxia or hypoxia for 24 h were subjected to immunoblot analysis with anti-HIF1α or anti-β-actin antibody. By densitometry, relative amounts of HIF1α protein normalized to β-actin were quantitated. *p < 0.05. **c** Total cell lysates from PASMCs exposed to normoxia or hypoxia for 24 h were subjected to immunoblot analysis with antibody against pS6K1, S6K1 or β-actin. By densitometry, relative amounts of phosphorylated S6K1 protein normalized to total S6K1 were quantitated. *p < 0.05 **d** Representative microphotographs of Ki-67 immunostaining of PASMCs exposed to normoxia or hypoxia, and calculation of Ki-67 index. Approximately 200 cells from at least 10 independent fields were counted for each condition, and Ki-67-positive cells are presented as a percentage of the total population. Scale bar represents 50 μm. *p < 0.05

To ascertain the effect of hypoxia on VSMCs, PASMCs exposed to normoxia or hypoxia for 24 h were prepared. We first examined the expression levels of genes known to be regulated by hypoxia, including VEGF, glucose transporter 1 (GLUT-1), and type I collagen prolyl-4-hydroxylase (C-P4H(I)) as well as miR-210 by qRT-PCR. The levels of all three gene transcripts and of mature miR-210 in PASMCs were increased by hypoxia (Fig. 1a). The protein level of HIF1α, a key transcription factor under hypoxic conditions, was then examined by immunoblotting. As expected, significant induction of HIF1α upon hypoxia was confirmed (Fig. 1b).

While hypoxia inhibits the mTOR signaling pathway in the majority of cells, mTOR signaling is known to be activated in the hypoxia-mediated VSMC proliferation phenotype [4]. To demonstrate that hypoxia mediates activation of the mTOR signaling pathway in PASMCs, we examined the phosphorylation of S6K1 (Thr389) in hypoxia-exposed PASMCs. The phosphorylation of S6K1 at threonine 389 (pS6K1) has been used as a hallmark of mTOR signaling pathway activation. Consistent with the results of previous studies [4], the phosphorylation of S6K1 (Thr389) was significantly increased by hypoxia (Fig. 1c).

Next, cell proliferation was examined. PASMCs exposed to normoxia or hypoxia were stained with an antibody against Ki-67 to measure the number of proliferating cells under each condition. Hoechst dye was used for nuclear staining. Approximately 9% of cells are Ki-67-positive under normoxia, and the percentage of Ki-67-positive cells increased to approximately 25% under hypoxia. Quantitative analysis of Ki-67 staining indicates that hypoxia increases the number of proliferating cells approximately 2.8-fold compared with normoxia control (Fig. 1d).

### Hypoxia regulates TSC1, a negative regulator of mTOR signaling

To understand the underlying molecular events leading to mTOR signaling pathway activation in response to hypoxia, we investigated the global mRNA profiles of PASMCs (normoxia) and their changes under hypoxia using NGS-based RNA sequencing (https://www.ncbi.nlm.nih.gov/geo/query/acc.cgi?acc=GSE118363). Library of RNA sequencing was made of total RNA from 6 x 10^5^ PASMCs and one sample per each condition was sequenced. From the RNA sequencing data, 302 genes were upregulated and 167 genes were downregulated upon hypoxia when differentially expressed genes (DEG) were adjusted to │fold change│≥ 2 (Supplementary data 1). KEGG pathway analysis for the DEG showed that genes involved in metabolic pathways were enriched. Clustered heatmap of KEGG pathway enrichment analysis is shown in Supplementary data 2.

**Fig. 2:**
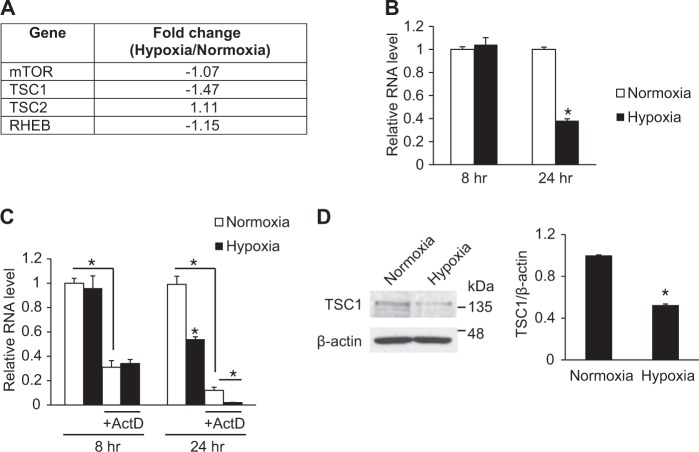
Hypoxia downregulates TSC1. **a** Expression levels of components of the mTOR signaling pathway, such as mTOR, TSC1, TSC2 and RHEB, were compared in PASMCs exposed to normoxia or hypoxia by NGS-based RNA sequencing. **b** Expression levels of TSC1 mRNA normalized to 18S rRNA were examined by qRT-PCR in PASMCs exposed to normoxia or hypoxia for 8 h or 24 h. *p < 0.05. **c** PASMCs were treated with 25 ng/ml actinomycin D for 2 h prior to hypoxia exposure for 8 h or 24 h. Level of TSC1 transcripts relative to 18S rRNA was measured by qRT-PCR. *p < 0.05. **d** Total cell lysates from PASMCs exposed to normoxia or hypoxia for 24 h were subjected to immunoblot analysis with anti-TSC1 or anti-β-actin antibody. By densitometry, relative amounts of TSC1 protein normalized to β-actin were quantitated. *p < 0.05

Among known key components of the mTOR signaling pathway such as mTOR, TSC1, TSC2 and RHEB, we observed that the relative expression level of TSC1 was significantly reduced to 68% in hypoxia-exposed PASMCs (Fig. 2a). No other genes investigated showed significant changes in hypoxic conditions. TSC1 mRNA levels were examined after exposure of hypoxia by qRT-PCR (Fig. 2b). The level of TSC1 transcripts was significantly reduced to 38% after 24 h of hypoxia in accordance with the RNA sequencing data, but the transcript level did not change after 8 h of hypoxia, suggesting that the downregulation of TSC1 by hypoxia is likely through post-transcriptional regulation. To test this possibility, PASMCs were treated with the RNA polymerase II inhibitor actinomycin D (ActD) prior to hypoxia exposure, followed by qRT-PCR analyses of TSC1 transcripts (Fig. 2c). After actinomycin D treatment to block new transcription, the amount of TSC1 transcripts was significantly reduced. TSC1 mRNA levels in cells exposed to hypoxia for 8 h did not change compared to normoxia in both presence and absence of actinomycin D. In contrast, the TSC1 transcript level was reduced by hypoxia exposure for 24 h and this downregulation occurred whether or not actinomycin D was added, indicating that existing TSC1 transcripts was downregulated by hypoxia. Therefore, this downregulation of TSC1 is likely to occur at the post-transcriptional level. The significant reduction of TSC1 protein levels by hypoxia was validated by immunoblotting (Fig. 2d). TSC1 is known to inhibit the mTOR signaling pathway in complex with TSC2. Therefore, these results suggest that the mTOR signaling pathway is activated upon hypoxia by downregulation of TSC1, a negative regulator of the mTOR signaling pathway.

### TSC1 regulates the proliferation of VSMCs through the mTOR signaling pathway

**Fig. 3:**
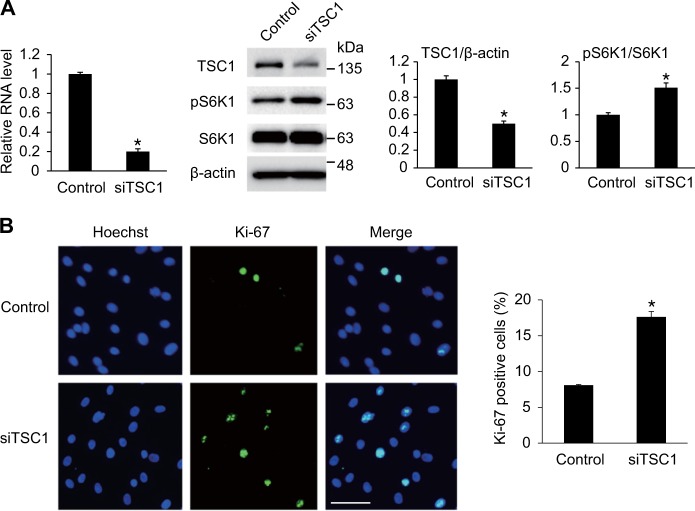
Regulation of mTOR signaling by TSC1 affects VSMC proliferation. **a** (Left panel) Expression levels of TSC1 mRNA normalized to 18S rRNA were examined by qRT-PCR in PASMCs transfected with control or siRNA against TSC1 (siTSC1) for 24 h. *p < 0.05. (Right panel) Total cell lysates from PASMCs transfected with control or siTSC1 for 24 h were subjected to immunoblot analysis with antibody against TSC1, pS6K1, S6K1 or β-actin. By densitometry, relative amounts of TSC1 protein normalized to β-actin and phosphorylated S6K1 protein normalized to total S6K1 were quantitated. *p < 0.05. **b** Representative images of Ki-67 immunostaining of PASMCs transfected with control or siTSC1, and calculation of Ki-67 index. Approximately 200 cells from at least 10 independent fields were counted for each condition, and Ki-67-positive cells are presented as a percentage of the total population. Scale bar represents 50 μm. *p < 0.05

Next, we confirmed that TSC1 affects the proliferation of PASMCs by regulating mTOR signaling. First, the effects of TSC1 on mTOR signaling activation in PASMCs were examined by downregulating TSC1 using siRNA (Fig. 3a). When TSC1 mRNA and protein levels were downregulated by siRNAs by approximately 80 and 50%, respectively, the phosphorylation of S6K1 (Thr389) increased by approximately 1.5-fold compared with control. This suggests that TSC1 plays a role as a negative regulator of mTOR signaling in PASMCs, as expected. We then examined whether cell proliferation was affected by the downregulation of TSC1. PASMCs transfected with siRNA against TSC1 were stained with a Ki-67 antibody to measure the number of proliferating cells (Fig. 3b). The percentage of Ki-67-positive cells increased approximately 2-fold when TSC1 was downregulated, suggesting that the downregulation of TSC1 is sufficient to promote the proliferation of PASMCs. All of these results indicate that the proliferation of VSMCs is regulated by modulating TSC1, a negative regulator in mTOR signaling.

### miRNA levels are regulated by hypoxia

Generally, miRNAs play a role in the negative post-transcriptional regulation of target genes. Multiple miRNAs have been proposed as important mediators modulating the VSMC phenotype [18, 19]. In addition, growing evidence suggests that a subset of miRNAs is up- or downregulated by hypoxia, and it was named “hypoxamirs” [20, 21] . Thus, we hypothesized that miRNAs modulated by hypoxia in VSMCs downregulate TSC1 post-transcriptionally by targeting TSC1 mRNA, leading to the activation of mTOR signaling. To search for miRNAs regulated by hypoxia in VSMCs, comprehensive miRNA profiles from PASMCs under normoxia and hypoxia were obtained by NGS-based small RNA sequencing (https://www.ncbi.nlm.nih.gov/geo/query/acc.cgi?acc=GSE118363). Library of small RNA sequencing was made of total RNA from 6 x 10^5^ PASMCs and one sample per condition was sequenced. We identified 23 miRNAs upregulated ≥ 1.5-fold in hypoxia-exposed PASMCs in comparison to those cells exposed to normoxia. The expression level of the master hypoxamir, miR-210, increased by approximately 3-fold in hypoxia-exposed PASMCs consistently with previous studies [22]. We also identified eight miRNAs whose expression is reduced to less than 50% under hypoxia (Table 1).

**Table 1:** miRNAs regulated by hypoxia from NGS analysis

miRNA	Fold change (Hypoxia/Normoxia)
hsa-miR-210-3p	3.69
hsa-miR-210-5p	3.20
hsa-miR-1260a	2.54
hsa-miR-1260b	2.54
hsa-miR-665	2.30
hsa-miR-1268a	2.16
hsa-miR-1268b	2.11
hsa-miR-5701	1.84
hsa-miR-33b-5p	1.77
hsa-miR-654-3p	1.70
hsa-miR-21-3p	1.63
hsa-miR-126-5p	1.63
hsa-miR-874-3p	1.59
hsa-miR-1185-2-3p	1.57
hsa-miR-431-5p	1.56
hsa-miR-485-3p	1.56
hsa-miR-378c	1.55
hsa-miR-1271-5p	1.55
hsa-miR-212-3p	1.52
hsa-miR-337-5p	1.52
hsa-miR-410-3p	1.51
hsa-miR-92b-3p	1.50
hsa-miR-766-3p	1.50
hsa-miR-3179	-11.54
hsa-miR-184	-7.70
hsa-miR-6859	-3.55
hsa-miR-19b-3p	-2.36
hsa-miR-19a-3p	-2.26
hsa-miR-1302	-2.25
hsa-miR-6511a	-2.16
hsa-miR-186-3p	-2.12

### miR-92b-3p is upregulated by hypoxia

**Fig. 4:**
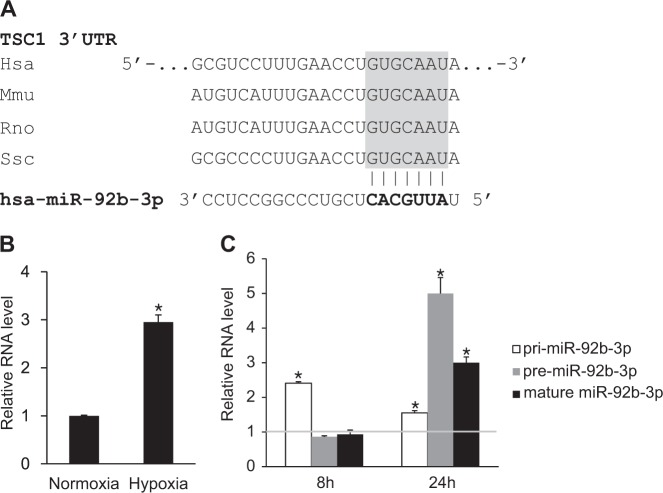
Hypoxia regulates the expression level of miR-92b-3p. **a** The miR-92b-3p MRE sequence found in the 3′-UTR of TSC1 mRNA is evolutionarily conserved. Hsa: human, Mmu: mouse, Rno: rat, Ssc: pig. **b**. Expression levels of miR-92b-3p normalized to U6 snRNA were examined by qRT-PCR in PASMCs exposed to normoxia or hypoxia for 24 h. *p < 0.05. **c** pri- and pre-miR-92b-3p levels relative to 18S rRNA and mature miR-92b-3p levels relative to U6 snRNA were measured by qRT-PCR at 8 h and 24 h after exposure to hypoxia. Data represent fold change of the RNA levels in hypoxia-exposed PASMCs from normoxia control cells. *p < 0.05

To investigate a possible interaction between miRNA and TSC1, we searched for potential miRNA recognition elements (MREs) of 23 miRNAs upregulated by hypoxia within the 3′-UTR of TSC1 gene using the TargetScan target prediction algorithm and the miRWalk database. We observed an evolutionarily conserved MRE partially complementary to miR-92b-3p in the 3′-UTR of the TSC1 gene (Fig. 4a). It suggests that miR-92b-3p might mediate reduced expression of TSC1 by direct targeting. Before testing whether miR-92b-3p targets TSC1, we validated the hypoxia-mediated induction of mature miR-92b-3p expression level by qRT-PCR (Fig. 4b). The expression of mature miR-92b-3p was induced approximately 3-fold after exposure to hypoxia for 24 h.

To understand at which biosynthetic step hypoxia regulates miR-92b-3p expression, we examined the levels of the primary transcript (pri-miR-92b-3p) and the precursor (pre-miR-92b-3p) as well as mature miR-92b-3p at 8 h and 24 h after exposure to normoxia or hypoxia (Fig. 4c). Expression levels of pri-miR-92b-3p, pre-miR-92b-3p and mature miR-92b-3p were all increased by hypoxia at the 24 h time point, but only pri-miR-92b-3p expression was induced by hypoxia at the 8 h time point, suggesting that miR-92b-3p is likely to be transcriptionally induced by hypoxia.

### TSC1 is a direct target of miR-92b-3p

**Fig. 5:**
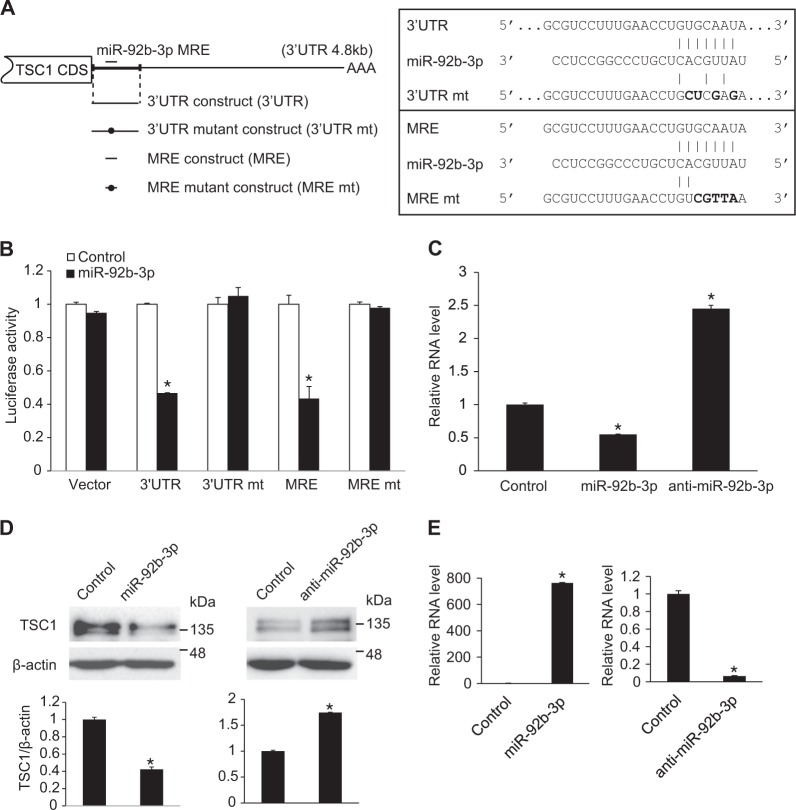
TSC1 is a target of miR-92b-3p. **a** (Left panel) Schematic diagram of predicted miR-92b-3p MRE in the 3′-UTR of TSC1 transcripts and luciferase reporter constructs used for luciferase assays. CDS and AAA stand for protein coding sequence and poly(A) tail, respectively. Mutations introduced in the MRE to disrupt a base paring with miR-92b-3p sequence are indicated as black circles. (Right panel) Sequences of the 3′-UTR, the 3′-UTR mt, the wild-type miR-92b-3p MRE in the 3′-UTR of TSC1 (MRE) and the MRE mt cloned into the 3′-UTR of the luciferase gene are shown. The mutated sequence is in bold. Perfect base matches are indicated by a line. **b** Luciferase activity of constructs, such as the 3′-UTR, the 3′-UTR mt, the MRE and MRT mt, were examined in Cos7 cells by transfecting control or miR-92b-3p mimic. A luciferase vector without 3′-UTR sequence (Vector) was used as a negative control. Data represent the mean ± S.E. of triplicates. *p < 0.05. **c**. Levels of endogenous TSC1 mRNA relative to 18S rRNA were quantified by qRT-PCR analysis in PASMCs transfected with control mimic, miR-92b-3p mimic, or anti-miR-92b-3p. Data represent the mean ± S.E. of triplicates. *p < 0.05. **d** Immunoblot analysis of TSC1 and β-actin using cell lysates of PASMCs transfected with control mimic, miR-92b-3p mimic or anti-miR-92b-3p were performed. Protein bands were quantitated by densitometry, and relative amounts of protein normalized to β-actin are presented. *p < 0.05. **e** Levels of endogenous miR-92b-3p relative to U6 snRNA were quantified by qRT-PCR analysis in PASMCs transfected with control mimic, miR-92b-3p mimic, or anti-miR-92b-3p. Data represent the mean ± S.E. of triplicates. *p < 0.05

To determine whether miR-92b-3p targets TSC1 by direct binding, we measured the expression of a luciferase reporter construct containing the 3′-UTR of *TSC1* in the presence of the miR-92b-3p mimic. Given that only one MRE of miR-92b-3p in the full length of the TSC1 3′-UTR (4884 bp) was predicted by computer algorithms, the *TSC1* 3′-UTR construct was generated with a part of TSC1 3′-UTR (935 bp) including the predicted MRE downstream of the luciferase reporter gene (Fig. 5a). The luciferase activity of the 3′-UTR construct was significantly reduced upon overexpression of the miR-92b-3p mimic, but not that of the 3′-UTR mutant construct which is the same construct with a mutated MRE region (Fig. 5b). This suggests that miR-92b-3p suppresses TSC1 expression by targeting the MRE within the 3′-UTR. To further support this notion that miR-92b-3p targets TSC1 through the evolutionarily conserved MRE in the 3′-UTR of the *TSC1* gene, a luciferase reporter construct containing the sequence of 23-bp MRE (MRE construct) was generated (Fig. 5a). miR-92b-3p reduced the luciferase activity of the MRE construct by approximately 60%. Mutations in the MRE construct (MRE mt), which disrupted base pairing with miR-92b-3p, abrogated the inhibition of luciferase activity by miR-92b-3p, suggesting that the conserved MRE is a critical target site for recognition of *TSC1* mRNA by miR-92b-3p (Fig. 5b).

We investigated whether endogenous TSC1 mRNA levels were modulated by miR-92b-3p in PASMCs. The level of TSC1 mRNA relative to 18s rRNA is reduced to approximately 50% in miR-92b-3p mimic-transfected PASMCs compared with the control miRNA-transfected PASMCs, indicating that miR-92b-3p downregulates TSC1 expression (Fig. 5c). Conversely, when miR-92b-3p was inhibited by the transfection of miR-92b-3p antisense inhibitor RNA (anti-miR-92b-3p) in PASMCs, TSC1 expression increased approximately 2.5-fold, indicating that the endogenous miR-92b-3p represses TSC1 expression. Moreover, immunoblot analyses indicated that the endogenous TSC protein level in PASMCs was reduced by exogenous miR-92b-3p mimic, but the basal level of TSC1 protein was elevated by anti-miR-92b-3p in comparison with control (Fig. 5d). All of these results demonstrate that TSC1 is a novel target of miR-92b-3p. To confirm overexpression and downregulation of miR-92b-3p, the level of miR-92b-3p was measured in PASMCs at 24 h after transfection with miR-92b-3p mimic or anti-miR-92b-3p (Fig. 5e).

### miR-92b-3p-mediated downregulation of TSC1 is essential for the activation of mTOR signaling upon hypoxia

**Fig. 6:**
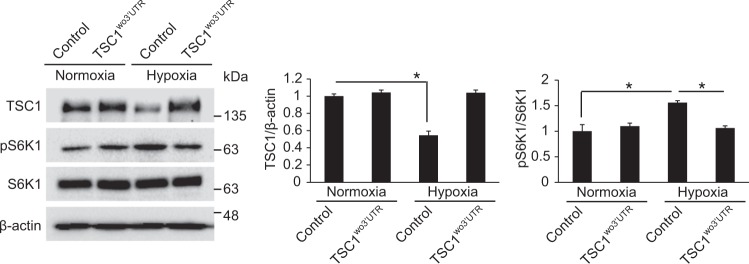
miR-92b-3p-mediated inhibition of TSC1 modulates mTOR signaling. PASMCs transfected with TSC1-expressing vector containing the miR-92b-3p binding site (Control) or TSC1 expression construct carrying the TSC1 cDNA deleted in the 3′-UTR (TSC1^wo3^′^UTR^) were exposed to normoxia or hypoxia for 24 h. Total cell lysates were subjected to immunoblot analysis with antibody against TSC1, pS6K1, S6K1 or β-actin. By densitometry, relative amounts of TSC1 protein normalized to β-actin and phosphorylated S6K1 protein normalized to total S6K1 were quantitated. *p < 0.05

To investigate the significance of miR-92b-3p-mediated downregulation of TSC1 on hypoxia-induced mTOR signaling activation, we examined whether mTOR signaling activation upon hypoxia is reversed by miR-92b-3p-refractory TSC1. We overexpressed exogenous *TSC1* mRNAs, which have deleted 3′-UTR and are resistant to miR-92b-3p, in PASMCs using Nucleofector (Lonza) and examined the changes in mTOR signaling activation under hypoxia by western blotting of phosphorylated S6K1 (Thr389) (Fig. 6). The TSC1-expressing vector containing the miR-92b-3p binding site within 3′-UTR was used as a control. Hypoxia-induced phosphorylation of S6K1 (Thr389) was significantly impaired when the exogenous 3′-UTR-deleted *TSC1* mRNAs (TSC1^wo3’-UTR^) are overexpressed, suggesting that miR-92b-3p-mediated downregulation of TSC1 is essential for the activation of mTOR signaling upon hypoxia.

### miR-92b-3p is responsible for the mTOR signaling pathway activation and VSMC proliferation

**Fig. 7:**
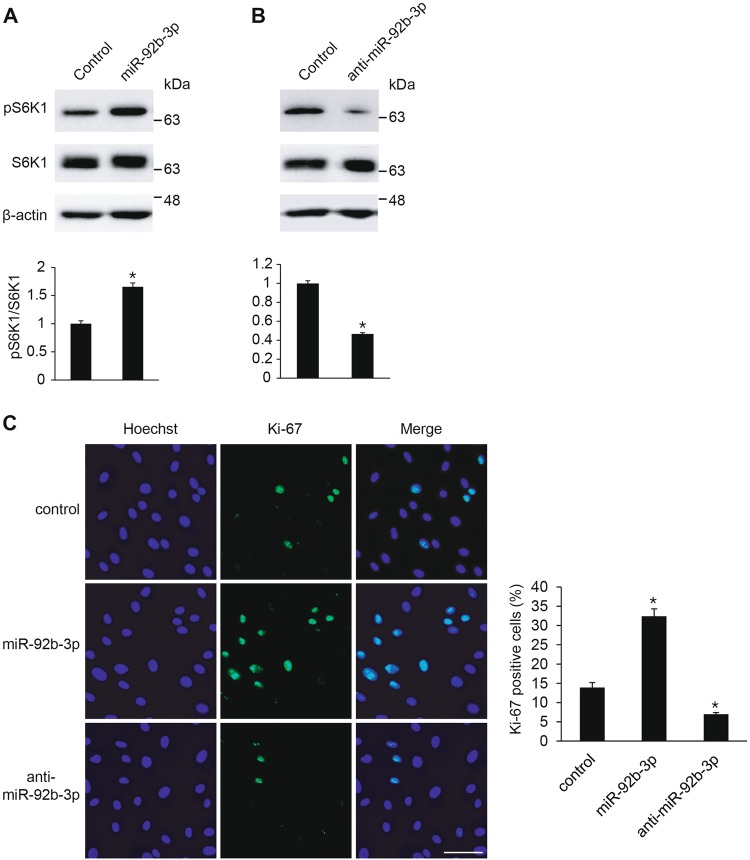
miR-92b-3p regulates mTOR signaling and VSMC proliferation. **a**, **b** Total cell lysates from PASMCs transfected with control mimic, miR-92b-3p mimic (**a**), or anti-miR-92b-3p (**b**) were subjected to immunoblot analysis with antibodies against pS6K1, S6K1 or β-actin. By densitometry, relative amounts of phosphorylated S6K1 protein normalized to total S6K1 were quantitated. *p < 0.05. **c** Representative microphotographs of Ki-67 immunostaining of PASMCs transfected with control mimic, miR-92b-3p or anti-miR-92b-3p, and calculation of Ki-67 index. Approximately 200 cells from at least 10 independent fields were counted for each condition, and Ki-67-positive cells are presented as a percentage of the total population. Scale bar represents 50 μm. *p < 0.05

To further support that miR-92b-3p regulates the mTOR signaling pathway by targeting TSC1 in VSMCs, we investigated the role of miR-92b-3p in regulation of the mTOR signaling pathway. PASMCs were transfected with miR-92b-3p mimic or anti-miR-92b-3p and subjected to immunoblot analysis using an antibody against phosphorylated S6K1 (Thr389) to examine activation of the mTOR signaling pathway. When miR-92b-3p was overexpressed in PASMCs, phosphorylation of S6K1 (Thr389) was induced (Fig. 7a). In contrast, anti-miR-92b-3p impaired the phosphorylation of S6K1 (Thr389) in PASMCs (Fig. 7b). These results suggest that miR-92b-3p acts as a positive regulator of the mTOR signaling pathway.

Next, the role of miR-92b-3p in the proliferation of VSMCs was examined (Fig. 7c). PASMCs transfected with control mimic, miR-92b-3p mimic or anti-miR-92b-3p were stained with an antibody against Ki-67. miR-92b-3p mimic significantly increased the number of proliferating cells approximately 2.3-fold in comparison with control. On the other hand, cells transfected with anti-miR-92b-3p showed significantly reduced numbers of Ki-67-positive proliferating cells by 50% in comparison with control. These results demonstrate that induction of miR-92b-3p is required for promoting the proliferation of VSMCs. Therefore, it is likely that miR-92b-3p induced by hypoxia promotes VSMC proliferation through activation of the mTOR signaling pathway by suppressing TSC1.

### Modulation of miR-92b-3p controls mTOR signaling-mediated VSMC proliferation induced by hypoxia

**Fig. 8:**
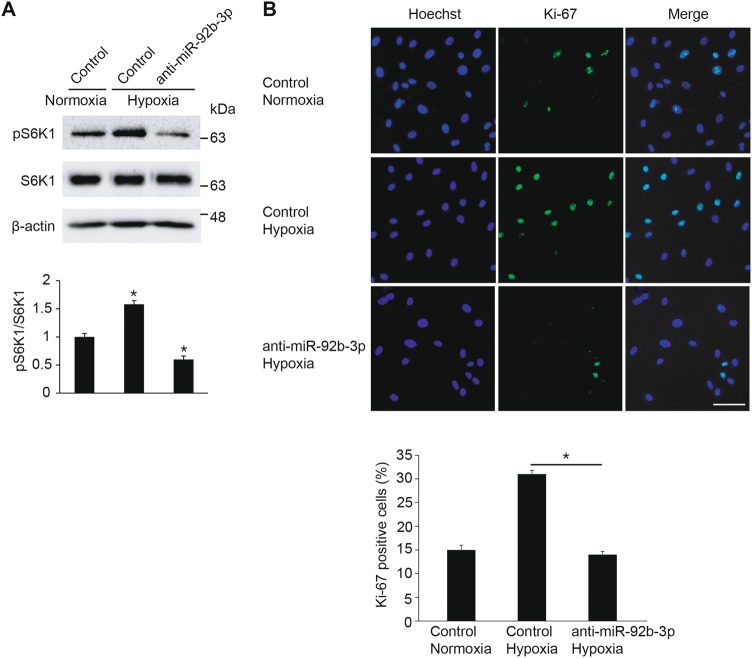
Modulation of miR-92b-3p controls mTOR signaling-mediated VSMC proliferation induced by hypoxia. **a** PASMCs transfected with control mimic or anti-miR-92b-3p were exposed to normoxia or hypoxia for 24 h. Total cell lysates were subjected to immunoblot analysis with antibody against pS6K1, S6K1 or β-actin. Relative amounts of phosphorylated S6K1 protein normalized to total S6K1 were quantitated by densitometry. *p < 0.05. **b** Representative microphotographs of Ki-67 immunostaining. PASMCs transfected with control mimic, miR-92b-3p or anti-miR-92b-3p were exposed to normoxia or hypoxia and then subjected to immunofluorescence staining with anti-Ki-67 antibody. Approximately 200 cells from at least 10 independent fields were counted for each condition, and Ki-67-positive cells are presented as a percentage of the total population. Scale bar represents 50 μm. *p < 0.05

Since hypoxia-induced miR-92b-3p activates the mTOR signaling pathway, we examined whether modulation of miR-92b-3p using anti-miR-92b-3p affects the hypoxia-mediated mTOR signaling activation (Fig. 8a). PASMCs transfected with control or anti-miR-92b-3p for 24 h were exposed to normoxia or hypoxia for 24 h. In control-transfected PASMCs, hypoxia accelerates the phosphorylation of S6K1 (Thr389) consistent with previous data (Fig. 1c). However, the hypoxia-induced phosphorylation of S6K1 (Thr389) was impaired when miR-92b-3p was downregulated by anti-miR-92b-3p, suggesting that modulation of miR-92b-3p controls the mTOR signaling pathway activation by hypoxia.

Next, we determined whether modulation of miR-92b-3p influences hypoxia-induced VSMC proliferation (Fig. 8b). PASMCs transfected with control mimic, miR-92b-3p or anti-miR-92b-3p were stained with an antibody against Ki-67. The percentage of proliferating cells was increased by hypoxia approximately 2-fold in comparison with control. However, when PASMCs were transfected with anti-miR-92b-3p prior to hypoxia exposure, the number of proliferating cells was significantly reduced to the level of control mimic-transfected normoxia cells. It suggests that downregulation of miR-92b-3p inhibits hypoxia-induced VSMC proliferation. Together, all the data suggest that modulation of miR-92b-3p controls mTOR signaling-mediated VSMC proliferation induced by hypoxia.

## Discussion

Hypoxia causes vasoconstriction of the pulmonary vasculature in concert with vascular remodeling leading to pulmonary hypertension [23]. Under hypoxia conditions, the proliferation of VSMCs is enhanced and activation of the mTOR signaling pathway is involved. Recent studies strongly suggest that miRNAs act as critical mediators of the hypoxic response [20, 21]. We thus hypothesized their prominent role in hypoxia-exposed VSMCs and aimed to characterize the overall miRNA expression profile in VSMCs by an NGS-based approach to identify hypoxia-modulated miRNAs. Twenty-three upregulated miRNAs were identified in hypoxia-exposed VSMCs compared with normoxia controls. Among the most significant changes observed in miRNAs with potential relevance to the mTOR signaling pathway was elevated miR-92b-3p. We demonstrated that hypoxia-induced miR-92b-3p targets a negative regulator in the mTOR signaling pathway, TSC1. Regulation of the miR-92b-3p-TSC1 axis is critical for hypoxia-induced proliferation of VSMCs. Inactivation of miR-92b-3p using an antisense inhibitor RNA reverses hypoxia-induced activation of the mTOR signaling pathway and cell proliferation.

miR-92b-3p has been reported to be involved in cell proliferation [24, 25]. For example, miR-92b-3p is enriched in human embryonic stem cells and controls their proliferation [26]. Moreover, miR-92b-3p has been shown to be upregulated in many types of human cancer and to promote cell proliferation and metastasis. Disabled homolog 2-interacting protein (DAB2IP), Smad7, reversion inducing cysteine rich protein with Kazal motifs (RECK), Dikkopf-3 (DKK3) and Smad3 have been identified as targets of miR-92b-3p and responsible for the promotion of cell proliferation and invasion in bladder cancer, hepatocellular carcinoma, osteosarcoma, glioma and glioblastoma, respectively [24, 25, 27–29]. However, these known target mRNAs were not downregulated in hypoxia-exposed VSMCs from our NGS-based RNA sequencing data (Table 2), suggesting that hypoxia-induced miR-92b-3p regulates the proliferation of VSMCs by targeting unknown novel targets. We identified a negative regulator in the mTOR signaling pathway, TSC1, as a novel target of miR-92b-3p in VSMCs. Upon hypoxia, TSC1 is suppressed by miR-92b-3p, which might inhibit TSC1/2 complex formation and lead to mTORC1 activation, resulting in mTOR signaling pathway activation and consequent increase in VSMC proliferation.

**Table 2:** Expression levels of known target mRNAs of miR-92b-3p from NGS analysis

Gene	Fold change (Hypoxia/Normoxia)
DAB2IP	1.04
Smad7	1.28
RECK	1.17
DKK3	1.16
Smad3	-1.07

Regulation of the mTOR signaling pathway by miRNAs has been studied [30]. Especially, a number of miRNAs have been identified to directly target components of the mTOR signaling pathway, because deregulation of multiple elements of the mTOR signaling pathway has been reported in many types of cancers [6]. For example, miR-99a, miR-99b, miR-100, miR-199a-3p, miR-224, miR-101-2, miR-544, miR-144-3p, miR-520c and miR-373 have been shown to target mTOR in colorectal cancer, lung cancer, cervical cancer, gastric cancer, breast cancer, hepatocellular carcinoma, salivary adenoid carcinoma, and fibrosarcoma cells [15, 31–39]. In addition, Rictor, a key component of the mTOR complex, has been reported as a target of miR-142-3p and miR-218 in lymphoma and oral cancer cells [40, 41]. However, the biological role of miR-92b-3p has not yet been reported in the context of mTOR signaling pathway regulation. In this study, we demonstrated a link between miR-92b-3p and the mTOR signaling pathway.

To date, miR-100 and miR-761 have been illustrated to have the role of an mTOR modulator in the regulation of vascular cell proliferation [42, 43]. An miRNA transcriptome analysis of hind-limb ischemia-induced mice demonstrated that miR-100 is downregulated after induction of ischemia. Potential target genes downregulated by miR-100 overexpression were screened for miRNA recognition elements in the 3′-UTR, and mTOR was identified as an miR-100 target gene in endothelial cells. More recently, miR-761 was identified as an mTOR targeting miRNA by screening potential miRNAs that target mTOR using a computer prediction algorithm, TargetScan. miR-761 repressed mTOR expression and consequently suppressed VSMC proliferation. Indeed, miR-761 is downregulated during angiotensin II-induced proliferation of VSMCs. We examined whether these miRNAs are downregulated in hypoxia-exposed PASMCs. From our NGS-based small RNA sequencing data, the level of miR-100 expression was not changed in response to hypoxia, and the expression of miR-761 was not determined. It suggests that it is unlikely that miR-100 and miR-761 are responsible for the mTOR signaling-mediated promotion of VSMC proliferation under hypoxia. We identified miR-92b-3p as a novel hypoxamir, and observed its role in the mTOR signaling-mediated proliferative response of hypoxic VSMCs.

Because the mTOR signaling pathway is involved in the increase of VSMC proliferation, which is a major pathological phenotype in hypoxia-induced vascular proliferative diseases, the mTOR signaling pathway could be considered a target for the treatment of vascular diseases including pulmonary artery hypertension. In this study, hypoxia-induced activation of the mTOR signaling pathway and proliferation in VSMCs are reversed by downregulation of miR-92b-3p using an antisense inhibitor RNA. This result suggests not only a molecular mechanism of miRNA-mediated mTOR signaling pathway regulation in hypoxia-induced VSMC pathogenesis but also a potential therapeutic strategy to effectively control development of vascular diseases.

## Materials and methods

### Cell culture and hypoxia

Human primary pulmonary artery smooth muscle cells (PASMCs) were purchased from Lonza (CC-2581) and were maintained in Sm-GM2 medium (Lonza) containing 5% fetal bovine serum (FBS). For hypoxia, the cells were placed in fresh medium and incubated in a sealed modular incubator chamber (Billups-rothenberg inc.) for 24 h at 37° C after flushing with a mixture of 5% CO_2_, 1% O_2_ and 94% N_2_ for 4 min.

### Quantitative reverse transcriptase-PCR (qRT-PCR)

Quantitative analysis of the change in expression levels was performed using real-time PCR. The mRNA levels were normalized to 18S rRNA. The primers used were as follows: TSC1, 5′-CTCCACAGCCAGATCAGACA-3′ and 5′-GCTGCCTGTTCAAGAACTCC-3′; VEGF, 5′-AAGGAGGAGGGCAGAATCAT-3′ and 5′-ATCTGCATGGTGATGTTGGA-3′; GLUT-1, 5′-CTTCACTGTCGTGTCGCTGT-3′ and 5′-CCAGGACCCACTTCAAAGAA-3′; C-P4H(I), 5′-AAGGCGAGATTTCTACCATA-3′ and 5′-TTGGTCATCTGAAGCAGACT-3′; 18S rRNA, 5′-GTAACCCGTTGAACCCCATT-3′ and 5′-CCATCCAATCGGTAGTAGCG-3′; pri-miR-92b-3p, 5′-CGGCGGATCTTTGATAGACT-3′ and 5′-GGAGGTGCTGGATGGAGTTA-3′. For quantification of mature miRNAs such as miR-92b-3p and miR-210, and pre-miR-92b-3p, the miScript PCR assay kit (Qiagen) and miScript precursor assay kit (Qiagen) were used, respectively, according to the manufacturer's instructions. Data analysis was performed using a comparative C_T_ method in the Bio-Rad software. miRNA levels were normalized to U6 small nuclear RNA. Three experiments were performed in triplicate, and the average results with standard errors are presented.

### miRNA mimics and anti-miRNA oligonucleotides

Chemically modified double-stranded RNAs designed to mimic the endogenous mature miR-92b-3p, miR-92b-3p antisense inhibitor RNA (anti-miR-92b-3p) and negative control miRNA were purchased from Genolution Pharmaceuticals. The miRNA mimics and anti-miRNA oligonucleotides were transfected at 5 nM and 50 nM, respectively, using RNAi Max (Invitrogen) according to the manufacturer's protocol.

### RNA interference

Small interfering RNA (siRNA) duplexes were synthesized by Genolution Pharmaceuticals. The target nucleotide sequence for TSC1 siRNA (5′- UAUUUAACAACAUCAGCCGUU-3′) was used. Negative control siRNA (Qiagen) was used as a control.

### Luciferase reporter constructs

A part of the 3′-UTR sequence of *TSC1* (935 bp) including the predicted miRNA recognition element (MRE) was cloned into the pIS0 vector (Addgene) containing the luciferase gene (3′-UTR). RT-PCR was used to amplify the 3′-UTR sequence of TSC1 from mRNA isolated from PASMCs using 5′-ATGGAGCTCTGTGTGGAAATGGGACGGAG-3′ and 5′-ATGGGCCGGCCTGGGAAATGATGGTCA-3′. For the 3′-UTR mt, which is the same 3′-UTR with a mutated MRE sequence, an upstream region and a downstream region of the MRE site in the 3′-UTR construct were amplified using primers including the *Xho*I enzyme recognition sequences. Two PCR products were digested by *Xho*I enzyme, ligated to change the MRE sequence into the *Xho*I recognition sequence and then cloned into the pIS0 vector. 5′-ATGGAGCTCTGTGTGGAAATGGGACGGAG-3′ and 5′-TCACTCGAGCAGGTTCAAAGGACGCAAC-3′ were used for the amplification of the upstream region. 5′-TCACTCGAGATGAGGCCAAATTTAATCTTTG-3′ and 5′-ATGGGCCGGCCTGGGAAATGATGGTCA-3′ were used for the amplification of the downstream region. The predicted MRE sequence (23 bp) and MRE mutant (MRE mt) sequence were cloned into the pIS0 vector (Addgene). 5′-GCGTCCTTTGAACCTGTGCAATACCGG-3′ and 5′-TATTGCACAGGTTCAAAGGACGCAGCT-3′ were used for the MRE and 5′-GCGTCCTTTGAACCTGTCGTTAACCGG-3′ and 5′-TTAACGACAGGTTCAAAGGACGCAGCT-3′ were used for the MRE mt.

### Luciferase assay

Cos7 cells were cotransfected with 5 nM miR-92b-3p or control mimic and luciferase reporter constructs using Lipofectamine 2000 (Life technologies). A β-galactosidase expression plasmid was used as an internal transfection control. Twenty-four hours later, luciferase assays were performed, and luciferase activity was presented after normalization to β-galactosidase activity.

### Immunoblotting

Cells were lysed in TNE buffer (50 mM Tris-HCl (pH 7.4). 100 mM NaCl. 0.1 mM EDTA) and total cell lysates were separated by SDS-PAGE, transferred to PVDF membranes, immunoblotted with antibodies and visualized using an enhanced chemiluminescence detection system (Amersham Biosciences). The antibodies used for immunoblotting were an anti-p70 S6 kinase (49D7), phospho-p70 S6 kinase (Thr389) and TSC1 (D43E2) from Cell signaling. An anti-β-actin antibody (sc47778) was purchased from Santa Cruz.

### Immunofluorescence staining

Equal amounts of PASMCs were seeded in chamber well slides and then exposed to hypoxia or transfected with control mimic, miR-92b-3p or anti-miR-92b-3p. Cells were fixed in 2% paraformaldehyde, blocked in 3% BSA in PBS and permeabilized in 0.1% Triton X-100 in PBS. The slides were sequentially probed with rabbit anti-human Ki-67 antibody (Abcam, #ab16667) and goat anti-rabbit IgG (H+L) cross-adsorbed secondary antibody, alexa flour 488 (Thermo Fisher Scientific, #A-11008). Nuclei were stained with Hoechst 33342 (Thermo Fisher Scientific, #62249). The slides were imaged by a Zeiss Axio Imager Z1 microscope. At least 2000 cells were counted per condition, and the percentages of Ki-67-positive cells were presented. The results are the mean ± S.E. for triplicate assays.

### TSC1 expression plasmids

Expression plasmid of TSC1 transcript with deleted 3′-UTR, pcDNA3.1 myc TSC1 (Addgene plasmid # 12133), was a gift from Cheryl Walker [44, 45]. To generate TSC1-expressing vector containing the miR-92b-3p binding site (Control), the pcDNA3.1 myc TSC1 was cleaved by the enzyme *Afl*II. Then, a PCR product including the 3′-UTR sequence (935bp) of *TSC1* was ligated into the cleaved pcDNA3.1 myc TSC1 plasmid. 5′-TACCTTAAGTGTGTGGAAATGGGACGGAG-3′ and 5′- ATACTTAAGGGCCTGGGAAATGATGGTCA-3′ were used for the PCR amplification. The TSC1 expression plasmids were transfected into PASMCs using P1 Primary Cell 4D-Nucleofector^TM^ X kit (Lonza) according to the manufacturer's protocol.

### NGS-based RNA sequencing

Total RNA was isolated from PASMCs exposed to normoxia or hypoxia for 24 h using Trizol (Invitrogen) according to the manufacturer’s instructions. cDNA libraries were constructed with the TruSeq RNA library kit using 1 μg of total RNA. The protocol consisted of polyA-selected RNA extraction, RNA fragmentation, random hexamer-primed reverse transcription, and 100-nt paired-end sequencing by Illumina HiSeq2000. The libraries were quantified using quantitative polymerase chain reaction (qPCR) according to the qPCR quantification protocol guide, and qualified using an Agilent Technologies 2100 Bioanalyzer. To estimate expression levels, the RNA-Seq reads were mapped to the human genome using TopHat [46] and determined using Cufflinks software [47]. The relative transcript abundances were measured in Fragments Per Kilobase of exon per Million fragments mapped (FPKM) using Cufflinks.

To identify altered gene expression levels, transcripts with at least one zeroed FPKM value across all samples were excluded from the analysis. We added 1 to each FPKM value to facilitate log2 transformation. Filtered data were logarithm-transformed and normalized by the quantile method.

### NGS-based small RNA sequencing

cDNA libraries were constructed with the small RNA library kit using 3 μg of total RNA from PASMCs. To generate a library product, adapter ligation, reverse transcription, PCR amplification, and pooled gel purification were conducted. The RNA 3′-adapter is specifically modified to target miRNAs and other small RNAs that have a 3′-hydroxyl group resulting from enzymatic cleavage by Dicer or other RNA processing enzymes. The adapters are ligated to each end of the RNA molecule and an RT reaction is used to create single stranded cDNA. The cDNA is then PCR amplified using a common primer and a primer containing one of 48 index sequences. The introduction of an index sequence at the PCR step separates the indexes from the RNA ligation reaction. To verify the size of the PCR enriched fragments, the template size distribution was checked by running on an Agilent Technologies 2100 Bioanalyzer using a DNA 1000 chip. The prepared libraries were quantified using qPCR according to the Illumina qPCR quantification protocol guide. Then, 50-nt single-end sequencing was performed by Illumina HiSeq2000.

After removal of the adaptor sequence in the reads, all reads were clustered to find unique sequences and those were then counted. In order to identify miRNA sequences, the unique sequences found after clustering were searched on the miRBase database by blast. Trimmed reads were considered as miRNA on the condition that they possess 100% sequence identity.

miRNAs with at least one zeroed read count value across all samples were excluded from the analysis. We added 1 to each count value to facilitate log2 transformation. Filtered data were logarithm-transformed and normalized by the TMM method.

### Statistical analysis

For each of the assays, three experiments were performed in triplicate, and the results were presented as the average with standard error. Statistical analyses were performed by an analysis of variance followed by Student’s *t* test using Prism 4 software (GraphPAD Software Inc.). *P* values of < 0.05 were considered significant and are indicated with asterisks.

## Electronic supplementary material

Supplemental data 1

Supplemental data 2
