# Supplementary figures and images for: miR-92b-3p-TSC1 axis is critical for mTOR signaling-mediated vascular smooth muscle cell proliferation induced by hypoxia
Source: Cell Death Differ. 2018 Dec 5;26(9):1782–95. doi: 10.1038/s41418-018-0243-z (PMC6748132; doi:10.1038/s41418-018-0243-z)

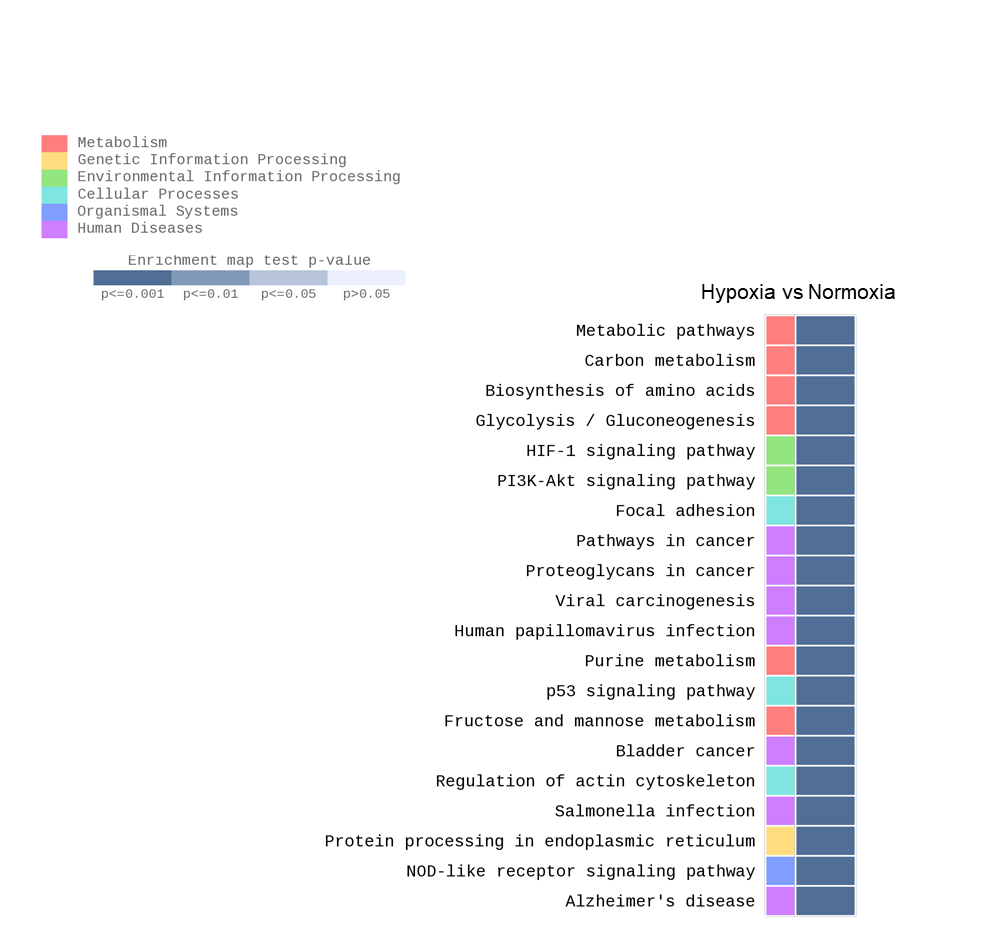

Supplement: Supplementary file 2 — Supplemental data 2 [file 41418_2018_243_MOESM2_ESM.png]
